# Catalytic Performance of Ni/CeO_2_/X-ZrO_2_ (X = Ca, Y) Catalysts in the Aqueous-Phase Reforming of Methanol

**DOI:** 10.3390/nano9111582

**Published:** 2019-11-08

**Authors:** Daniel Goma, Juan José Delgado, Leon Lefferts, Jimmy Faria, José Juan Calvino, Miguel Ángel Cauqui

**Affiliations:** 1 Departamento de Ciencia de los Materiales e Ingeniería Metalúrgica y Química Inorgánica, Universidad de Cádiz, 11510 Puerto Real, Spain; 2 IMEYMAT, Instituto de Microscopía Electrónica y Materiales, 11510 Puerto Real, Spain; 3 Catalytic Processes and Materials group, University of Twente, P.O. Box 217, 7500 AE Enschede, The Netherlands

**Keywords:** aqueous-phase reforming, nickel, ceria, zirconia, calcium, yttrium, methanol

## Abstract

In this study, we reported on the effect of promoting Ni/ZrO_2_ catalysts with Ce, Ca (two different loadings), and Y for the aqueous-phase reforming (APR) of methanol. We mainly focused on the effect of the redox properties of ceria and the basicity provided by calcium or yttrium on the activity and selectivity of Ni in this reaction. A systematic characterization of the catalysts was performed using complementary methods such as XRD, XPS, TPR, CO_2_-TPD, H_2_ chemisorption, HAADF-STEM, and EDS-STEM. Our results reveal that the improvement in reducibility derived from the incorporation of Ce did not have a positive impact on catalytic behaviour thus contrasting with the results reported in the literature for other Ce-based catalytic compositions. On the contrary, the available Ni-metallic surface and the presence of weak basic sites derived from Ca incorporation seem to play a major role on the catalytic performance for APR of methanol. The best performance was found for a Ce-free catalyst with a molar Ca content of 4%.

## 1. Introduction

The future of the so-called hydrogen economy is linked to the possibility of developing economical clean and sustainable methods for both the production of H_2_ and its subsequent conversion into energy [1]. Some of the applications adapted to this energy model would operate, for example, with electricity generated in a fuel cell powered by in situ produced hydrogen thus overcoming problems associated with storage, transport, and handling of hydrogen gas [2]. In this sense, methanol derived from biorefinery water fractions can be considered an interesting source of hydrogen, as it is an economic product with a considerable H_2_ content (13%) and easily transportable (liquid at room temperature). The transformation of methanol into hydrogen can be achieved by reforming, with the aqueous-phase reforming (APR) proposed by Dumesic [3] being the most interesting alternative as long as it is carried out at low temperature and does not require prior vaporization of the reagents such as in the case of classic steam reforming processes [4].

The APR of methanol involves the decomposition of the organic compound to produce CO and H_2_ (Equation (1)). In this case, C–C bond cleavage is not necessary, and CO and H_2_ are produced through C–H and O–H bond cleavage. The CO is then converted into CO_2_ and H_2_ by the water–gas shift (WGS) reaction, for which H_2_O dissociation is needed (Equation (2)).

CH_3_OH → CO + 2H_2_ (1)

CO + H_2_O → CO_2_ + H_2_ (2)

However, under normal operating conditions (i.e., low temperature), the hydrogenation reactions of CO and CO_2_ to produce methane and water (Equations (3) and (4)) are thermodynamically favoured, compromising the H_2_ selectivity in APR.

CO + 3H_2_ → CH_4_ + H_2_O (3)

CO_2_ + 4H_2_ → CH_4_ + 2H_2_O (4)

Thus, a suitable catalyst for APR should promote the reforming (Equation (1)) and WGS (Equation (2)) reactions and inhibit the methanation reactions (Equations (3) and (4)).

Davda et al. [5] reported a comparative study of the catalytic behaviour of a series of metals such as Pt, Ru, Rh, Pd, Ir, and Ni in APR reactions. The catalytic activities obtained for Pt and Ni were comparable and superior to those shown by the rest of the metals. Although nickel catalysts are economically preferable to Pt, they have two major drawbacks for APR: (1) they favour the methanation reactions therefore reducing the hydrogen selectivity [6,7] and (2) they have a significant tendency to deactivation by oxidation or sintering under hydrothermal conditions [8]. To become a real alternative to Pt in APR, these two limitations of Ni catalysts must be overcome. One of the most commonly used strategies consists of combining nickel with specific promoters or supports. Thus, for example, it has been reported that Ni/CeO_2_ shows great potential to be used in the APR of glycerol, exhibiting higher H_2_ selectivity than Ni/Al_2_O_3_ [9]. Improved catalytic performances were also obtained with Ni–Ce–O catalysts for aqueous-phase reforming of ethanol [10]. This is generally attributed to the singular redox properties of ceria (i.e., high oxygen storage capacity and oxygen mobility) which promote preferentially the WGS versus the methanation reaction and, thus, enhancing the hydrogen production [11,12]. The CeO_2_–ZrO_2,_ CeO_2_–La_2_O_3_ and CeO_2_–TiO_2_ mixed oxides have also been successfully used as support of Pt or Ni for APR, achieving a higher hydrothermal stability compared to pure CeO_2_ [13,14,15,16].

The effect of the basicity of the support has also been investigated by several authors. Guo et al. [17] found that the catalytic performances of a series of Pt-supported catalysts followed the same trend than the basicity measured by CO_2_-TPD. They concluded a correlation between the WGS ability, promoted in this case by basic supports, and the APR activity. Menezes et al. [18] reported that Pt/MgO performed better that Pt supported on alumina, zirconia or ceria, and attributed its success to the basicity of MgO.

In a previous work, we reported on the performance of a Ni/CeO_2_/YSZ (YSZ: yttrium-stabilized zirconia) catalyst for the CO_2_ reforming of CH_4_ [19]. This formulation exhibited not only high activity but also an outstanding stability under reaction conditions. The excellent redox properties provided by the support were found to be a key factor in preventing the accumulation of carbon deposits responsible for the deactivation of the catalyst. Based on that work, we here report on a series of Ni/CeO_2_/X-ZrO_2_ (X = Ca, Y) catalysts designed to simultaneously develop enhanced redox and basic properties, the latter resulting from the incorporation of calcium. These catalysts have been prepared and evaluated for hydrogen production by APR of methanol. The effect of these properties on the activity and selectivity of Ni has been specially addressed in order to determine their relative influence on the catalytic performance. This is a crucial point for tailoring more efficient catalysts for APR. A detailed characterization of the catalysts employing different techniques such as X-ray diffraction (XRD), X-ray photoelectron spectroscopy (XPS), temperature programmed reduction with H_2_ (H_2_-TPR), temperature programmed desorption of CO_2_ (CO_2_-TPD), high-angle annular dark-field scanning transmission electron microscopy (HAADF-STEM) and energy dispersive X-ray spectroscopy (EDS-STEM) was carried out to look for correlations between chemical properties and catalytic behaviour.

## 2. Materials and Methods

### 2.1. Catalysts Preparation

Calcia-stabilized zirconia (CSZ), yttria-stabilized zirconia (YSZ), and pure zirconia (Z) were used as supports for nickel and ceria-modified nickel catalysts. These oxides were prepared by a hydrothermal method from a 0.5 M zyrconil nitrate (ZrO(NO_3_)_2_·6H_2_O) (Sigma Aldrich, St. Louis, MO, USA) solution containing calculated amounts of calcium or yttrium nitrates (Sigma Aldrich, St. Louis, MO, USA) to obtain molar ratios of 4% and 14%, in the case of CSZ samples (4CSZ and 14CSZ), and 8% in the case of the YSZ (8YSZ). The pH was adjusted to 10 by adding a NaOH (Sharlau, Barcelona, Spain) 1 M solution under vigorous stirring. The hydrothermal synthesis was performed in PTFE vessels heated at 200 °C over 24 h. After cooling to room temperature, the powders were separated by centrifugation and washed with Milli-Q water until pH = 7. Subsequently, they were dried at 110 °C, grounded, sieved (300–600 µm fraction), and finally calcined at 500 °C for 1 h. Ceria (12% w/w) was incorporated by incipient wetness impregnation using a Ce(NO_3_)_3_·6H_2_O (Alfa Aesar, Kandel, Germany) 2 M aqueous solution. After drying at 110 °C and calcination at 500 °C (1 h), the samples were further impregnated using a Ni(NO_3_)_2_·6H_2_O (Sigma Aldrich, St. Louis, MO, USA) 2 M aqueous solution. After the second impregnation, the final powders were dried at 110 °C overnight and finally calcined at 500 °C (1 h). The nickel loading was always 6% w/w.

### 2.2. Catalysts Characterization

The metal contents of fresh and used catalysts were determined by inductively coupled plasma atomic emission spectroscopy (ICP-AES). The measurements were carried out using a Thermo Elemental plasma atomic emission spectrometer (model Intrepid, Thermo Scientific, Waltham, MA, USA).

The textural properties of catalysts were determined from nitrogen physisorption at −196 °C. The adsorption and desorption isotherms were obtained using a Quantachrome Autosorb IQ3 (Quantachrome Instruments, Boynton Beach, FL, USA). Prior to the physisorption experiments, the samples were degassed for 2 h at 200 °C under vacuum. Surface area was calculated with the BET method, and the pore volume and diameter were calculated by the BJH method using data from the desorption-isotherm.

The Ni-metallic surface area and average particle size were determined by hydrogen chemisorption. The experiments were performed at 35 °C in a ASAP 2020 equipment (Micromeritics Instrument Corp., Norcross, GA, USA). Prior to each adsorption, samples were reduced in 5% H_2_/Ar flowing at 750 °C for 1 h and evacuated under vacuum at the same temperature. Estimated metal surface area and particle size values were based on spherical geometry and an H/Ni = 1 adsorption stoichiometry.

The structure of catalysts was determined by X-ray diffraction (XRD) in a diffractometer D8 Advance (Brucker, Billerica, MA, USA) using nickel-filtered CuKα (λ = 0.15418 nm) radiation with a step size of 0.02° and 0.2 s as the counting time. The crystallite diameter for NiO and Ni species in calcined and reduced samples, respectively, was estimated applying the Scherrer equation (*k* = 0.9), using the DIFFRAC.EVA software from Brucker. The reduction treatment consisted of heating in a flow of 5% H_2_/Ar from room temperature to 750 °C (10 °C /min), followed by 1 h of isothermal treatment at this temperature. The gas was switched to He for 1 h, and then the samples were cooled down to −80 °C in He flow. Finally, they were heated from −80 °C up to room temperature under 5% O_2_/He to avoid overheating of the reduced catalysts when exposed to air.

The reducibility of the catalysts was studied by H_2_ temperature programmed reduction (H_2_-TPR), using Autochem 2920 II equipment (Micromeritics Instrument Corp., Norcross, GA, USA). Before the TPR experiments, samples were treated in 5% O_2_/He for 1 h at 500 °C, purged with He at the same temperature, and cooled down to room temperature. Then, they were heated (10 °C/min) from room temperature up to 950 °C in flowing 5% H_2_/Ar. Hydrogen consumption throughout the reduction process was monitored by means of a thermal conductivity detector (TCD).

Temperature programmed desorption of CO_2_ (CO_2_-TPD) experiments were carried out to characterize the surface basic sites. Prior to the analyses, the catalysts were reduced in situ under a 5% H_2_/Ar flow at 750 °C for 1 h, treated with He for an additional hour and cooled down to room temperature. Afterwards, they were contacted with a flow of pure CO_2_ for one hour at 100 °C and finally cooled down again and purged with He at room temperature before starting the TPD experiment. CO_2_ desorption was followed by monitoring the m/z = 44 signal using a quadrupole mass spectrometer model GSD301T1 (Pfeiffer Vacuum, Wetzlar, Germany). The calibration of this signal was carried out using standards of calcium oxalate diluted in alumina.

X-ray photoelectron spectroscopy (XPS) experiments were carried out in a Kratos Axis Ultra DLD (Kratos Analytical Ltd, Manchester, UK) equipped with a monochromatized Al-Kα X-ray source (1486.6 eV), operating with an accelerating voltage of 15 kV and 10 mA current. Spectra were acquired in a constant analyser energy mode, with a pass energy of 20 eV. Powder samples were analysed without any pre-treatment. Surface charging effects were corrected by adjusting the binding energy of the C(1s) peak at 248.8 eV. CasaXPS software (version 2.3.19) (Casa Software Ltd, Devon, UK) was used for the data analysis. Prior to the XPS experiments, the catalysts were reduced using the same protocol as that used for XRD measurements.

A JEOL-2010F microscope (Jeol Ltd., Tokyo, Japan) with a spatial resolution at Scherzer defocus conditions of 0.19 nm in TEM and operated at 200 kV was used in high-angle annular dark field scanning transmission electron microscopy (HAADF-STEM) mode. The microscope was equipped with an X-ray energy-dispersive spectrometer (X-EDS) model Xmax SSD (Oxford Instruments, Abingdon, UK), for composition analysis at the sub-nanometre scale. Samples for EM studies were prepared by depositing the powders onto holey carbon-coated Cu grids. Additionally, very high spatial resolution X-EDS maps were acquired using the ChemiSTEM capabilities of an FEI Titan Themis 60–300 microscope (FEI Company, Hillsboro, OR, USA).

Particle size distributions were obtained from the statistical analysis of at least 250 particles observed in the HAADF images. The values of mean particle size ($\bar{d\;}$) and surface area-weighted mean particle size (${\bar{d}}_{sa}$) were calculated according to Equations (5) and (6), respectively. For a suitable consideration of the contribution of the larger particles, Ni dispersion (D%) was not estimated from the mean particle size, but as the ratio of total surface to bulk Ni atoms. These, in turn, were estimated by accumulating the surface or bulk Ni atoms corresponding to each of the particles considered in the analysis (assuming spherical morphology).

(5) $$\bar{d\;}=\;\frac{\sum n_{i}\;d_{i}}{\sum n_{i}}\;$$

(6) $${\bar{d}}_{sa}=\;\frac{\sum n_{i}d_{i}^{3}}{\sum n_{i}d_{i}^{2}}$$

### 2.3. Catalytic Tests

Catalytic tests were carried out in a laboratory scale system as the one depicted in Figure S1 (Supporting Materials). The experiments were performed in a continuously operated stainless-steel tubular reactor with a cross-section of 6 mm using downward flow. The catalyst (without diluent) was loaded on the mid-section of the reactor on a fixed bed with a 5 μm pore mesh. Aqueous-phase reforming of methanol was carried out at 230 °C and 32 bar, using a flow rate of 0.338 mL/min of 5 wt. % aqueous solution of methanol as a feed (WSHV = 4 h^−1^). The selected reaction time was 5 h (after 90 min to reach steady state). 

The outlet stream from the reactor was cooled down, pressure-controlled by a back-pressure regulator, and separated (without dilution with inert gas) into the liquid and gas streams. The composition of the gas products was analysed in situ by means of a micro gas chromatograph (GC, Varian CP4900, equipped with MS5 and PPQ columns) (Varian Inc., Palo Alto, CA, USA) and the liquid products were taken every 30 min and analysed offline by performing HPLC (RID-10A detector, Aminex HPX-87H column, 300 × 7.8 mm) (Shimadzu Corporation, Kyoto, Japan). Prior to the catalytic tests, samples were reduced at 750 °C for 1 h under pure hydrogen. After catalytic tests, the used samples were recovered and weighted to evaluate the catalyst loss during reaction. Methanol conversion, selectivity, and H_2_ yield were obtained as follows:

(7) $${\%\;\mathrm{CH}}_{3}\mathrm{OH}\;\mathrm{conversion}=\frac{{\left[{\mathrm{CH}}_{3}\mathrm{OH}\right]}_{\mathrm{In}}-{\left[{\mathrm{CH}}_{3}\mathrm{OH}\right]}_{\mathrm{Out}}}{{\left[{\mathrm{CH}}_{3}\mathrm{OH}\right]}_{\mathrm{In}}}\times 100$$

(8) $$\%\;\mathrm{Selectivity}\;\mathrm{to}\;X=\frac{{\left[X\right]}_{\mathrm{Out}}}{{\left[H_{2}\right]}_{\mathrm{Out}}+{\left[{\mathrm{CO}}_{2}\right]}_{\mathrm{Out}}+{\left[\mathrm{CO}\right]}_{\mathrm{Out}}+{\left[{\mathrm{CH}}_{4}\right]}_{\mathrm{Out}}}\times 100$$

(9) $$H_{2\;}\mathrm{yield}\;\left(\%\right)=\frac{{\mathrm{mol}\;H}_{2}{}_{\mathrm{Out}}}{{\mathrm{mol}\;\mathrm{CH}}_{3}{\mathrm{OH}}_{\mathrm{In}}}x\;\frac{1}{3}\;x\;100$$

## 3. Results and Discussion

### 3.1. Catalysts’ Composition and Textural Characterization

The composition of the catalysts obtained from ICP analysis is shown in Table 1. As it can be seen, the elemental contents are in good agreement with the nominal values. Similar results were obtained in the analysis of the catalysts after reaction thus discarding the occurrence of lixiviation under reaction conditions (Table S1). 

The results obtained from the textural characterization of the catalysts using N_2_ adsorption–desorption isotherms (Figure S2) are also gathered in Table 1. Samples exhibited mesoporosity, with surface areas values varying in the range 30–67 m^2^⋅g^−1^. The lower values (around 30 m^2^⋅g^−1^) were obtained for the catalysts using pure zirconia as support. Those systems based on structurally stabilised zirconia (CSZ or YSZ) exhibited both higher S_BET_ and pore volume values. This effect has previously been explained in the literature as being due to the existence of defects in the material which would impede the enlargement of grain boundaries and, hence, prevent growth of crystalline aggregates [20].

The incorporation of Ce did not have a clear influence on the surface area of the final catalyst; however, it provoked a decrease in both pore volumes and average pore sizes which, in principle, suggests that ceria was mainly blocking the largest pores of the support.

### 3.2. Structural Analysis by X-Ray Diffraction (XRD)

**Figure 1:**
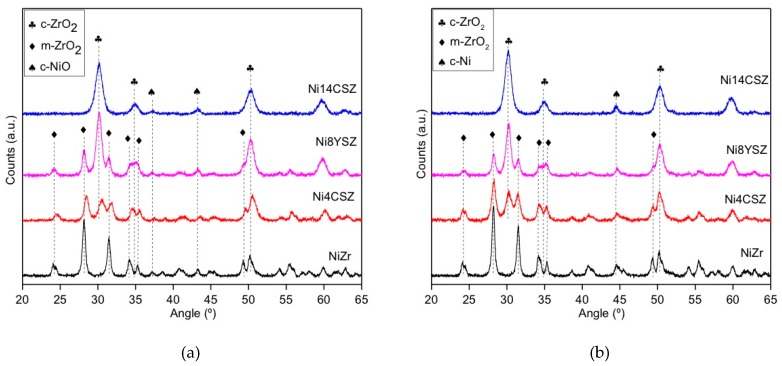
XRD patterns of the investigated catalysts (without Ce): (**a**) calcined; (**b**) reduced.

**Figure 2:**
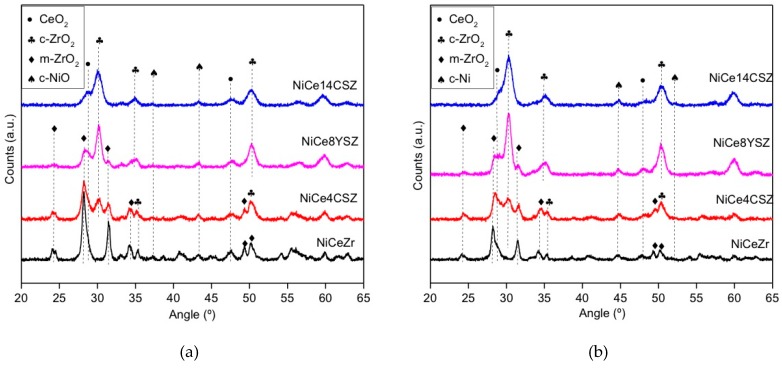
XRD patterns of the investigated catalysts (with Ce): (**a**) calcined; (**b**) reduced.

The XRD patterns of calcined and reduced catalysts are depicted in Figure 1 and Figure 2. As expected, all the diffractograms were dominated by diffraction peaks characteristic of the corresponding support. Thus, the catalyst supported on pure ZrO_2_ presented peaks associated to the monoclinic structure of this oxide (PDF No. 37-1484). Doping with Ca^2+^ or Y^3+^ led to the appearance of additional peaks, indicating the partial stabilization of the cubic/tetragonal structure of zirconia (PDF No. 24-1074 and 83-0113, respectively). The degree of stabilization was higher in the case of the sample with 8% of Y^3+^ with respect to the sample doped with 4% of Ca^2+^. We must recall that, according to the difference in oxidation states of Y and Ca, doping in these two samples introduced a similar concentration of oxygen vacancies in the ZrO_2_ network. Increasing the doping amount up to 14% of Ca^2+^ led to an almost complete structural stabilization of the cubic/tetragonal phase, as deduced from the disappearance of the diffraction peaks associated to the monoclinic form. No peaks corresponding to segregated Ca-containing species were observed.

After the incorporation of Ce (Figure 2), some additional broad peaks at 2θ values of 28.5°, 33.1°, 47.5°, and 56.3° appeared, indicating the presence of small crystals of ceria with its typical fluorite-like structure (PDF No. 34-0394).

**Figure 3:**
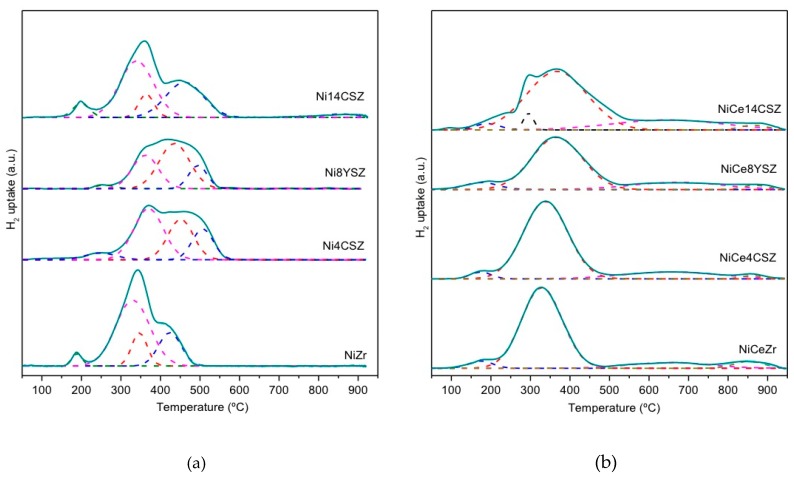
H_2_-TPR profiles: (**a**) samples without Ce; (**b**) samples with Ce.

As for the Ni, all the calcined samples showed very small peaks at 37.2° and 43.2°, accounting for the presence of NiO (PDF No. 47-1049). After reduction at 750 °C, these peaks transformed into those corresponding to metallic Ni (Figure 2 and Figure 3).

The average crystallite sizes of the NiO and Ni phases were estimated using XRD line-broadening and the Scherrer equation (Table 2). In particular, the peaks at 43.2° and 44.5° on the diffractograms corresponding to the calcined and reduced catalysts were used, respectively. Despite the low intensity of these peaks, the values obtained point to some influence of the support on the crystallite size of nickel species. In the case of the NiZr catalyst, values between 22 and 24 nm were obtained for NiO and Ni crystallites, respectively. The incorporation of either Ce or Ca to the catalysts slightly decreased the average crystallite size of nickel species, suggesting an improvement of the metal dispersion over the different Ce- or Ca-modified zirconia supports.

For samples containing ceria, the crystallite size for this phase was also estimated. As deduced from data in Table 2, the incorporation of Ca^2+^ or Y^3+^ into the zirconia support allows for the obtention of smaller (more dispersed) CeO_2_ crystallites. This effect can be due to the higher surface area of the doped samples with respect to pure zirconia but also to a better structural coherence between the fluorite-type structure of ceria and the cubic/tetragonal structure stabilised in doped-zirconia supports.

### 3.3. Hydrogen Chemisorption and Temperature Programmed Reduction (TPR) Studies

To gain more insight into the Ni dispersion on the different supports, hydrogen chemisorption measurements were carried out. The results obtained are summarised in Table 2 in terms of Ni-metallic surfaces and Ni-particle sizes. As it can be seen, similar values were obtained for Ni-metallic surfaces, being only slightly higher in the case of the samples containing 4% of Ca. It should be noted that the values for particle size estimated from H_2_ chemisorption were higher than those obtained by XRD (crystallite sizes). These fell around 20 nm, with a larger deviation in the case of the Ni14CSZ (38 nm) and Ni8YSZ (32 nm) catalysts. Results obtained from XRD line broadening were rather consistent with those obtained using electron microscopy, indicating that the estimations made from H_2_ chemisorption measurements were likely affected by Ni–support interactions. 

The TPR experiments were performed in order to obtain information about reducibility and also about Ni–support interactions in these catalysts. The reduction profiles are shown in Figure 3, and Table 2 includes the hydrogen consumption and estimated reduction degree values, assuming that, initially, Ce was completely Ce^4+^ and Ni as Ni^2+^. Values close to 100% were obtained indicating that full reduction of these two species, from Ce^4+^ to Ce^3+^ and from Ni^2+^ to Ni^0^, respectively, was achieved at the end of the TPR experiments. Values higher than 100% observed in some cases have also been reported by other authors for similar compositions, being generally attributed to (i) the existence of a small amount of Ni^3+^-forming non-stoichiometric NiO_1+x_ species [21,22] and/or (ii) the reduction of labile oxygen adsorbed on vacancies in the ZrO_2_ support generated as a consequence of doping with Ca^2+^ or Y^3+^ [23,24].

Concerning the structure of the TPR profiles, they were constituted by a combination of several peaks which account for the different reduction steps. For a better interpretation of the reduction sequence, the TPR profiles were deconvoluted into individual Gaussian functions as shown in Figure 2. These peaks are grouped into three general categories: low-temperature peaks (LT; 180–250 °C), intermediate-temperature peaks (IT; 300–500 °C), and high-temperature peaks (HT; >550 °C). The LT contributions were commonly low-intensity peaks assigned to the reduction of oxygen adsorbed on support vacancies and/or reduction of non-stoichiometric NiO_1+x_ species well dispersed on the catalyst surface [21,25]. This LT contribution appeared in the reduction scheme of all our catalysts, being slightly shifted to lower temperatures for catalysts containing Ce.

The IT part of the TPR profiles generally comprised several peaks which are commonly associated with the reduction of NiO species with different particle sizes and degrees of interaction with the support. Thus, it is widely assumed that NiO aggregates with little interaction with the substrate are reduced at temperatures around 400 °C while those with stronger interaction can be reduced at higher temperatures (approximately 550 °C) [26,27,28,29]. The reduction of large NiO aggregates in two consecutive steps (Ni^2+^ → Ni^δ+^ → Ni) within this IT range has also been proposed by different authors [24].

In the case of catalysts without Ce, at least two contributions (centred at approximately 350 °C and 450 °C, respectively) are required to obtain a good fit of the curves in this IT range. Considering that no significant differences in NiO crystallite sizes were observed by XRD, we can assume that the intensity of the peak at higher temperature is related to the existence of NiO particles having a stronger interaction with the support [26]. Note that in the case of Ni8YSZ and Ni4CSZ catalysts, the first contribution was less intense, while the second was slightly shifted at higher temperature, suggesting the occurrence of a stronger NiO–support interactions in theses samples.

On the contrary, for the Ce-containing samples, a single peak was observed in the IT region of the TPR profiles, with the only exception being the NiCe14CSZ catalyst, which also showed a sharp shoulder at 300 °C. These simpler profiles indicate that CeO_2_ improved the reducibility of the NiO phases in these catalysts. According to quantitative estimations, the amounts of H_2_ involved in the IT part of the TPRs were higher than those required for the complete reduction of Ni, indicating that not only Ni^2+^ but also Ce^4+^ species were being reduced simultaneously at these temperatures. High-temperatures peaks (>550 °C) also appearing in the TPR-profiles of Ce-containing samples would account for the reduction of bulk Ce species existing in larger (or not in contact with Ni) CeO_2_ particles.

In summary, the TPR results indicate that NiO species in these catalysts were reduced mainly in a temperature range between 300 and 500 °C. For the catalysts supported on 4CSZ and 8YSZ, a shift to higher temperatures was observed presumably as a consequence of a stronger NiO-support interaction occurring in these cases. The incorporation of Ce improves the reducibility of NiO which seems to occur in a single step and simultaneously with the reduction of most of the CeO_2_.

### 3.4. Thermal Programmed Desorption of CO_2_ Basicity Studies

**Figure 4:**
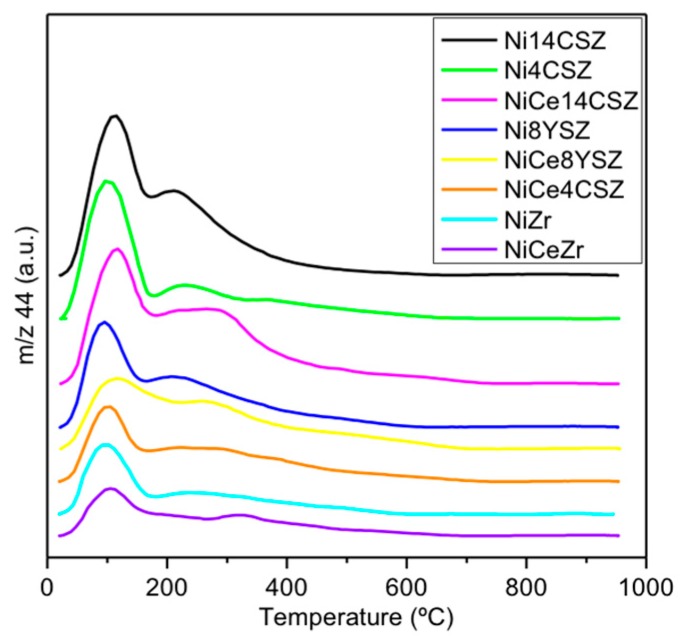
CO_2_ desorption profiles.

Thermal programmed desorption of CO_2_ (CO_2_-TPD) experiments were carried out to investigate the number and strength of surface basic sites. Desorption profiles are depicted in Figure 4. They were deconvoluted into three temperature ranges, as shown in Figure S3. The low temperature range (90–180 °C) contains information about weak basic sites; the intermediate temperature range (180–400 °C) gives information about moderate basic sites, and, finally, the high temperature range (>400 °C) accounts for the stronger basic sites. The amounts of CO_2_ desorbed in each range as well as the total amount of CO_2_ desorbed are gathered in Table 3.

As can be seen in Figure 4, the CO_2_-TPD profile of the NiZr sample showed a strong desorption peak at approximately 100 °C as well as a broad band extending up to 600 °C which can be deconvoluted into different contributions accounting for medium/strong basic sites. Taking this sample as a reference, we observed, as expected, that the incorporation of Ca results in a general increase in basicity [30], especially significant in the range of weak basic centres for the Ni4CSZ sample. When Y is used instead of Ca, the basicity with respect to the NiZr sample also increases, but more homogeneously for both weak- and intermediate-type centres. Thus, although the total amount of basic centres is almost the same for the Ni4CSZ and Ni8YSZ samples (25.6 and 26.0 µmol CO_2_⋅g^−1^, respectively), the latter exhibits fewer weak basic sites but more medium/strong basic centres. Increasing the calcium content by up to 14% results in an increase in the amounts of CO_2_ desorbed over the whole temperature range. The incorporation of Ce has a minimal effect on the surface basicity of the NiZr sample, but, on the contrary, it greatly influences not only the quantity but mainly the nature of the basic centres of Ca(Y)-doped supports. In general, after the incorporation of Ce, the intensity of the low temperature peak (weak basicity) decreases, probably due to the partial covering of the support surface by CeO_2_, while new contributions appeared in the intermediate/high temperature range, likely associated to the Ce-O sites [31]. 

### 3.5. Analysis of the Surface by XPS

**Figure 5:**
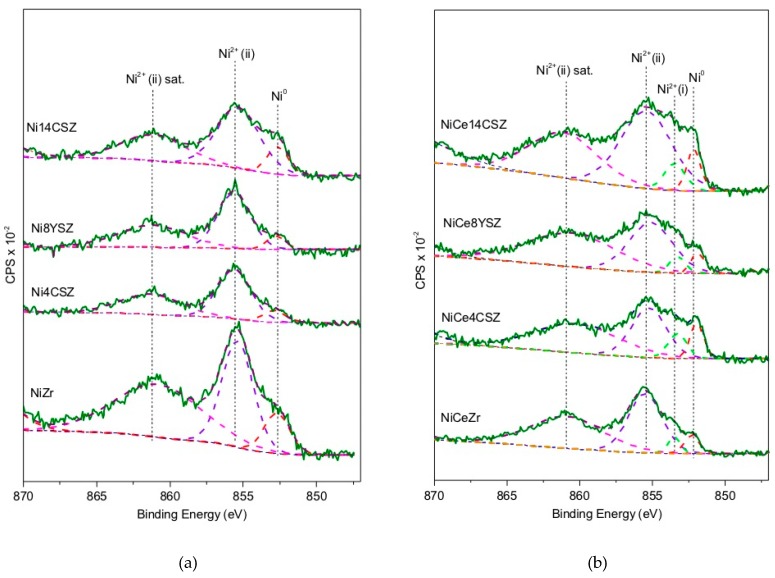
Nickel 2p XPS spectra: (**a**) samples without Ce; (**b**) samples with Ce.

X-Ray photoelectron spectroscopy was used to obtain information about the chemical state of elements on the surface of Ni catalysts after reduction treatment. Binding-energy values for Ni 2p, Ce 3d, and O 1s core levels are included in Table 4. Figure 5 gathers the core level Ni 2p XPS spectra corresponding to Ce-free and Ce-containing catalysts. The presence of Ni^0^ was confirmed in all cases by the peak centred at approximately 852 eV. Moreover, two peaks at 855 eV and 860 eV were also observed in all the spectra. According to the literature, they can be assigned to Ni^2+^ (as Ni(OH)_2_) and its satellite, respectively [32,33,34]. They have been labelled as Ni^2+^ (ii) in the spectra of Figure 5. The appearance of these peaks reveals a partial re-oxidation of the catalysts due to the exposure to atmospheric conditions during transfer from the preparation reactor to the XPS analysis chamber. It should be noted that, in the case of catalysts containing Ce, an additional component at around 853.5 eV, just in the middle of the positions corresponding to Ni^0^ (852.6 eV) and Ni^2+^ (NiO, 854.6 eV), was observed. This peak, identified as Ni^2+^(i) in the spectra, has been ascribed by some authors also to NiO [35]. However, its appearance exclusively in the case of Ce-containing samples suggests that it may be due to the cationic Ni^δ+^ species resulting of a Ni–Ce interaction [36] or simply Ni^2+^ incorporated into the CeO_2_ lattice at the surface level [37,38].

The cerium-reduction degree was calculated fitting the experimental Ce 3d profiles (Figure S4) with two reference spectra corresponding to samples with either 100% Ce^3+^ or 100% Ce^4+^ according to the procedure described in Reference [39]. As observed in Table 4, the addition of Ca significantly increased the Ce^3+^ percentage with respect to the CeZr substrate, but a maximum of 18%–19% was obtained independently of the Ca loading (4% or 14%). The highest amount of reduced Ce (34%) was obtained for the NiCe8YSZ catalyst, suggesting that the surface concentration of oxygen vacancies over ceria particles was also higher in this catalyst compared to those using Ca-stabilized ZrO_2_ supports.

From O 1s spectra (Figure S5), two types of oxygen species were found, with peaks at around 529 and 531 eV for all the catalysts. They can be ascribed to lattice oxygen and adsorbed OH^–^ groups as suggested in the literature [40,41,42].

### 3.6. Electron Microscopy Study of Ni Catalysts Using HAADF-STEM and EDS-STEM

**Figure 6:**
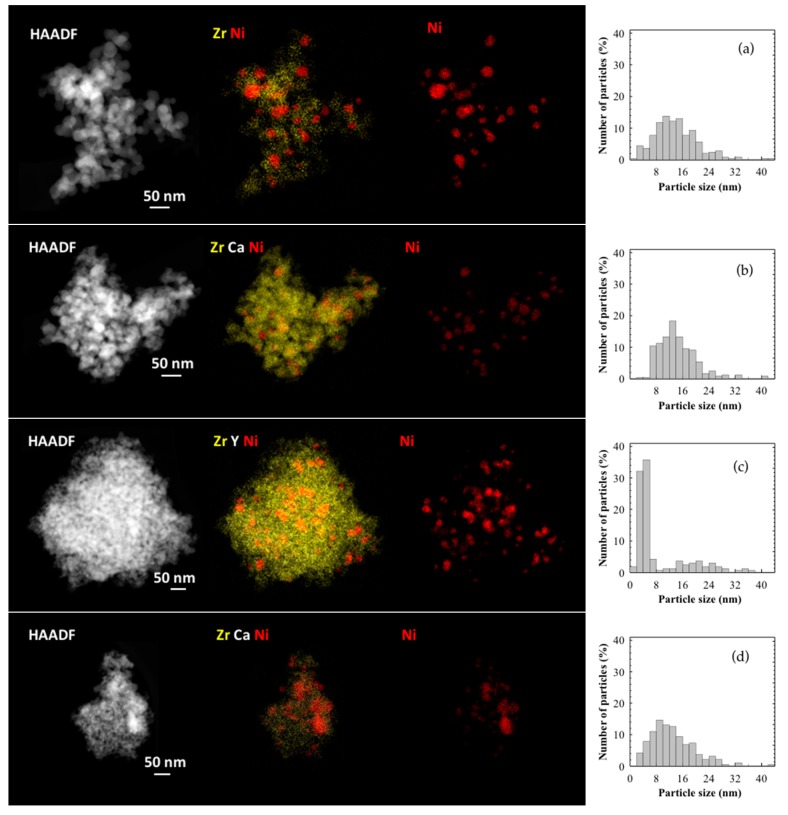

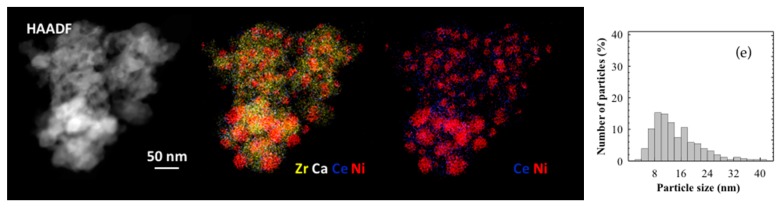
HAADF images, EDS mappings, and particle size distributions of: (**a**) NiZr; (**b**) Ni4CSZ; (**c**) Ni8YSZ; (**d**) Ni14CSZ; and (**e**) NiCe4CSZ.

Representative HAADF images recorded in STEM mode are shown in Figure 6. Moreover, and with the aim of exploring the spatial distribution of elements in the Ni catalysts, EDS analyses, also in STEM mode, were performed (for simplicity, only the images corresponding to NiZr, Ni4CSZ, Ni8YSZ, Ni14CSZ, and NiCe4CSZ are shown). As can be seen, the images and chemical maps indicate a rather homogeneous distribution of the different elements in all the investigated catalysts (maps for individual components are shown in Figure S6). 

Particle size distributions obtained from the statistical analysis of the particles observed in the HAADF images are also included in Figure 6. Data corresponding to mean particle size ($\bar{d\;}$), surface area-weighted mean particle size (${\bar{d}}_{sa}$), and Ni dispersion (D%) are gathered in Table 5. In the case of NiZr catalyst (Figure 6a), the EDS-STEM maps show that Ni particles were homogeneously distributed with a mean particle size of approximately 14 nm. A few particles with sizes in the range 40–84 nm were also observed. When the particle size distribution contains both very small and very large particles, the value of the surface area-weighted mean diameter is more representative in terms of metallic dispersion than that estimated from the mean particle size. As can be observed, the value of ${\bar{d}}_{sa}$ (27 nm) was similar to the averaged particle size estimated from H_2_ chemisorption (27 nm) or XRD (24 nm).

Values between 20 and 23 nm were obtained for the Ni4CSZ, Ni8YSZ, and Ni14CSZ catalysts, which were also close to the crystallite sizes obtained by XRD. According to the values obtained by HAADF, the incorporation of Ca and Ce did not seem to have a significant influence on Ni particle size as suggested by the XRD results.

The accumulated dispersion values resulting from the particle size distribution curves were also very similar (5%–6%), being only slightly higher in the case of samples containing Ca.

**Figure 7:**
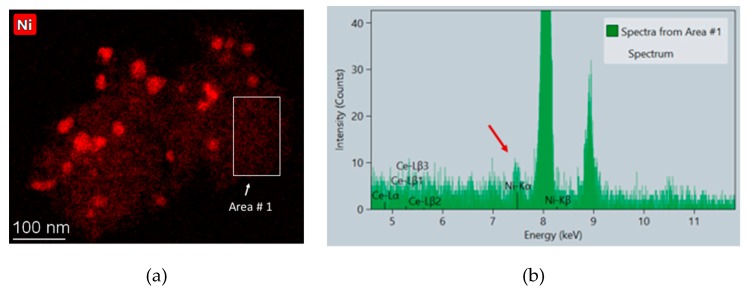
EDS analysis corresponding to Ni4CSZ: (**a**) Ni mapping; (**b**) spectrum from the area indicated in the image.

It must be pointed out that, due to the atomic number of Ni, very small nickel nanoparticles cannot be easily identified in HAADF-STEM images. In order to evaluate this issue, EDS-STEM analyses were carried out at high magnifications, selecting areas of the material in which apparently no metallic particles but only a background signal was observed. Figure 7 illustrates this type of analysis. As can be seen in the EDS spectrum of the selected area, the presence of Ni was clearly detected which confirms the existence of highly dispersed forms of Ni, whose contribution in the particle size distribution analysis is not considered. 

**Figure 8:**
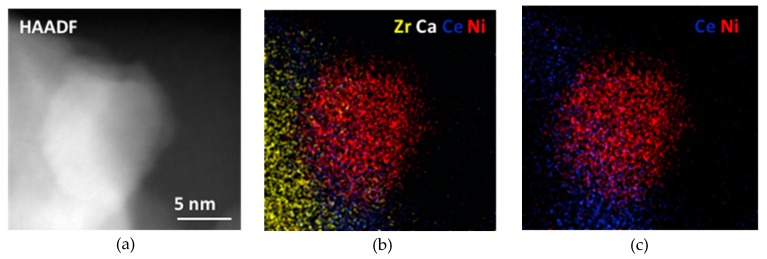
(**a**) HAADF image and (**b**,**c**) EDS mappings corresponding to the NiCe4CSZ catalyst.

This technique also allowed us to obtain information about the relative spatial distribution of the different components of the catalyst at the nanoscopic level which is an important issue to understand the interactions among them, mainly in the case of the CeO_2_-containing catalysts. As illustration, Figure 8 shows a detailed, high-magnification image of the NiCe4CSZ catalyst. As it can be clearly seen, CeO_2_ is in direct contact with Ni, forming a layer at the interface between the metallic particle and the support. The interaction resulting from this direct contact may be responsible for the improvement in the reducibility of Ni particles observed in the TPR profiles corresponding to the catalysts containing CeO_2_.

### 3.7. Catalytic Activity Tests

Table 6 summarizes the performance of Ni catalysts in the APR of methanol at 230 °C and 32 bar after 5 h on stream. A blank experiment was carried out with the 4CSZ support, showing no activity under the same experimental conditions. Unconverted methanol in the liquid phase and CO_2_, CO, H_2_, and CH_4_ in the gas phase were the only products found at the reactor outlet.

Conversions higher than 40% were found with all the investigated Ni catalysts. Taking the NiZr sample as a reference, we observed that the incorporation of Ca had a positive effect on methanol conversion, which reached the highest value (75%) for the Ni4CSZ catalyst. In contrast, doping of ZrO_2_ with Y had no significant impact on activity. As already mentioned, the doping of the zirconium oxide support with 4% Ca or 8% Y induced a stabilization of the tetragonal structure, generating in both cases a similar amount of oxygen vacancies. The different responses obtained with the Ni4CSZ and Ni8YSZ catalysts with respect to the NiZr sample suggests that there is not a straightforward relationship among these two parameters (oxygen vacancies concentration and APR activity). On the other hand, the conversion value obtained for the Ni14CSZ catalyst (63%) indicates that increasing the Ca content above 4% may have a negative effect on activity.

**Figure 9:**
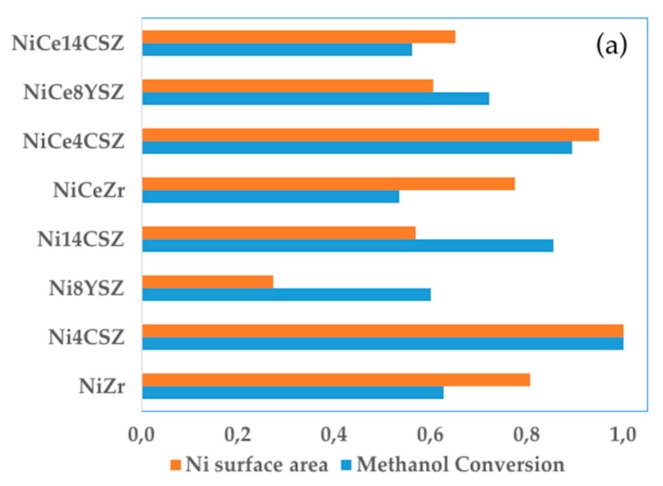

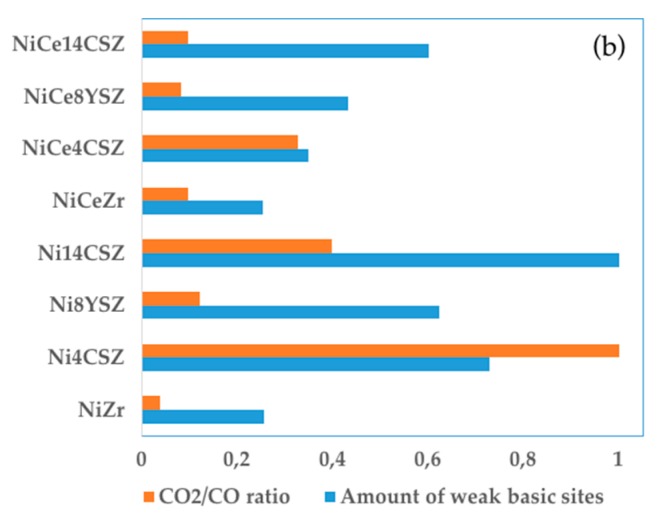
(**a**) Ni-surface area and methanol conversion (normalized values); (**b**): CO_2_/CO ratio and amount of weak basic sites (normalized values).

To explain the excellent behaviour exhibited by the Ni4CSZ sample, we should first consider its higher metallic surface area. For this purpose, we decided to compare the conversion and surface area of the different Ni catalysts. To facilitate the reading of the figure, we rationalized all the values with respect to the Ni4CSZ catalyst. As shown in Figure 9a, the higher conversions were obtained for Ni4CSZ and NiCe4CSZ which were also the catalysts with the larger Ni-surface areas. The metal surface is therefore a key factor determining the catalytic behaviour of these catalysts. However, the analysis of the whole set of values suggests that, in addition to the amount of Ni atoms exposed on the surface, some additional factor must be influencing the catalytic performance in this reaction.

One of the most significant differences resulting from the characterization of the catalysts was related to the surface basicity measured in the CO_2_-TPD experiments. The influence of the basicity of the support on the APR reaction has been investigated in the literature and it has been proposed that basic sites promote a water–gas shift and further enhance the APR process [17]. As commented in a previous section, the incorporation of Ca or Y into the ZrO_2_ lattice increased the total basicity of the samples (0.57 µmol CO_2_·g^−1^ for NiZr versus 1.07, 1.08, and 1.52 µmolCO_2_·g^−1^ for Ni4CSZ, Ni8YSZ, and Ni14CSZ, respectively). However, this increase in basicity did not have an identical incidence in all types of centres. Thus, in the case of the Ni4CSZ sample, the additional basicity was associated to the development of new weak basic sites, whereas, for Ni8YSZ, it resulted in an increase in the number of medium/strong centres. On the other hand, the situation for the Ni14CSZ sample can be described in terms of a simultaneous increase in both types of sites. The order of activity found for these samples suggests that weak centres have a positive influence on activity, while the influence of medium/strong sites seems to be negative for the APR reaction. The low metallic surface area of the Ni14CSZ may also be influencing its lower activity in comparison with Ni4CSZ.

Despite the differences found in methanol conversion, all catalysts showed very similar values of H_2_ selectivity, ranging from 73% to 76%. The H_2_/CO_2_ ratio was close to the stoichiometric value (H_2_/CO_2_ = 3) in the case of Ca-containing catalysts and slightly higher in the case of NiZr (3.6) and Ni8YSZ (3.5), confirming that the WGS reaction was less favoured in the latter catalysts. Similar conclusions can be derived from data corresponding to CO selectivity (0.2 and 0.5 for Ni4CSZ and Ni14CSZ, respectively) or the CO_2_/CO ratio.

To explain the influence of Ca in selectivity, we must recall that basic sites act promoting the transformation of CO to CO_2_ in the WGS reaction [17]. However, sites with a high basic strength may have a negative influence avoiding the desorption of CO_2_. The higher WGS activity showed by the Ni4CSZ catalyst could be related with the nature of the basicity exhibited by this sample, characterised by a high concentration of weak basic sites and a low concentration of medium/strong centres. The correlation between weak the basic sites and CO_2_/CO ratio is illustrated in Figure 9b.

As for the effect of Ce on the APR performance of Ni, a decrease in conversion was generally observed in the catalysts containing Ce with respect to the homologous series without Ce, with the unique exception of Ni8YSZ, for which methanol conversion increased from 46% to 54% after the incorporation of Ce. Moreover, the activity order observed for this series was as follows: NiCe4CSZ (68) > NiCe8YSZ (54) > NiCe14CSZ (44) > NiCeZr (40). These results reveal that the improvement in reducibility derived from the incorporation of Ce (and evidenced in the TPR experiments) did not have a direct impact on catalytic behaviour thus contrasting with the results reported in the literature for other Ce-based catalytic compositions. For example, it has been reported that cerium oxide promotes catalyst activity in WGS reaction due to the fact of its well-known oxygen storage capacity and oxygen mobility throughout the lattice [11,12]. In order to explain this apparent disagreement, we must recall that Ce is not only changing the redox properties of the catalysts but also their surface basicity. According to CO_2_ desorption measurements, new basic sites with intermediate strength appeared on catalyst surfaces after the addition of Ce. Simultaneously, the number of weaker basic sites decreased significantly, probably because these sites, initially located on the surface of the Ca(Y)–ZrO_2_ supports, resulted in being partially covered by the CeO_2_ layer. Changes in basic strength may be responsible for the decrease in methanol conversion after incorporation of Ce. These results also imply that the negative effects on APR of methanol resulting from changes in basicity prevail over the positive effects derived from changes in redox properties, both effects caused by the incorporation of Ce.

Another factor to be considered is the formation of cationic Ni species, presumably derived from the observed Ni–Ce interaction. These species, evidenced by XPS (peaks at around 853 eV) only in Ce-containing catalysts, would be inactive for the reaction. However, we must also bear in mind that reduction treatments prior to XPS measurements were not performed in situ and, therefore, this cationic species may result from air exposure of the pre-reduced catalysts.

As for selectivity, the most remarkable finding related with CeO_2_ is a decrease in CO production for the NiCeZr sample compared to that of NiZr which can be explained considering that both catalysts showed a very similar basicity and, therefore, both conversion and selectivity values would depend mainly on other factors affected by Ce (e.g., reducibility).

## 4. Conclusions

In this work, a series of Ni/ZrO_2_ catalysts were prepared, characterized, and tested in the aqueous-phase reforming of methanol reaction. The effects of doping zirconia with calcium or yttrium and the promotion with CeO_2_ were investigated. The best catalytic performance in terms of activity and selectivity was found for the catalyst doped with 4% molar of Ca. This catalyst showed a higher metallic area and developed surface basicity characterized by the presence of a high concentration of weak basic centres and a low concentration of medium/strong basic sites. These two factors seem to be responsible for its excellent catalytic performance. The incorporation of ceria has two significant effects on the properties of Ni catalysts: (i) improves reducibility and (ii) increases the strength of the basic sites. The lower activity of catalysts promoted with CeO_2_ suggests that the improvement in redox properties does not compensate for the negative effects on APR resulting from changes in surface basicity.

## Figures and Tables

**Table 1:** Composition and textural properties.

Catalysts	Composition from ICP (% w/w)					S_BET_ (m^2^·g^−1^)	Pore Volume (cm^3^·g^−1^)	Average Pore Radius (nm)
	Ni	Ce	Ca	Y	Zr			
NiZr	7.0	-	-	-	60.0	30	0.178	9.4
NiCeZr	5.9	12.6	-	-	48.4	30	0.141	7.7
Ni4CSZ	5.0	-	1.20	-	60.0	48	0.214	5.7
NiCe4CSZ	5.9	13.0	1.80	-	47.6	47	0.123	3.9
Ni8YSZ	5.6	-	-	4.30	56.4	53	0.192	5.7
NiCe8YSZ	5.5	12.7	-	3.62	46.1	42	0.123	3.9
Ni14CSZ	6.9	-	4.90	-	53.0	54	0.220	4.8
NiCe14CSZ	5.5	13.5	4.00	-	43.3	67	0.133	2.8

**Table 2:** Results from H_2_ chemisorption, XRD line broadening, and H_2_-TPR experiments.

Catalysts	Metal Surface ^1^ (m^2^·g^−1^)	Ni Particle Size ^1^ (nm)	Crystallite Size (nm) ^2^			H_2_ Uptake ^3^ (mmol·g^−1^)	Reduction Degree ^3^ (%)
			Ni	NiO	CeO_2_		
NiZr	1.7	27.0 ± 1.0	24	22	-	1.16	97
NiCeZr	1.7	24.5 ± 2.0	10	17	22	1.53	105
Ni4CSZ	2.2	15.6 ± 0.5	17	15	-	0.94	110
NiCe4CSZ	2.1	19.3 ± 1.2	11	17	13	1.63	110
Ni_8_YSZ	1.2	32.5 ± 3.3	19	21	-	0.93	97
NiCe8YSZ	1.3	24.0 ± 1.6	16	18	15	1.28	92
Ni14CSZ	1.2	38.0 ± 1.4	21	18	-	1.24	105
NiCe14CSZ	1.4	25.7 ± 1.2	16	15	10	1.69	118

^1^ From H_2_ chemisorption; ^2^ from XRD line broadening. Crystallite sizes for NiO and CeO_2_ were obtained from patterns corresponding to calcined catalysts, and, for Ni, they were obtained from patterns corresponding to the reduced catalysts. ^3^ From H_2_-TPR experiments.

**Table 3:** Amounts of CO_2_ desorbed in the CO_2_-TPD experiments. Values obtained by integration of the deconvoluted profiles in the low, medium and high temperature ranges.

Samples	Weak (90–180 °C) (µmol·g^−1^)	Intermediate (180–400 °C) (µmol·g^−1^)	Strong (>400 °C) (µmol·g^−1^)	Total (µmol·g^−1^)
NiZr	7.4	3.3	2.9	13.7
Ni4CSZ	21.1	1.5	3.1	25.6
Ni8YSZ	18.1	7.9	-	26.0
Ni14CSZ	29.0	7.4	-	36.5
NiCeZr	7.3	3.9	2.6	13.8
NiCe4CSZ	10.1	6.9	4.7	21.8
NiCe8YSZ	12.5	14.6	9.9	37.0
NiCe14CSZ	17.4	15.8	6.9	40.1

**Table 4:** Binding-energy values of main peaks and the Ce^3+^ percentage from XPS.

Samples	Ni 2p	Ce 3d	O 1s	Ce^3+^ (%)
NiZr	852.7, -, 855.4	-	529.5, 531.2	-
Ni4CSZ	852.9, -, 855.3	-	529.7, 531.7	-
Ni8YSZ	852.8, -, 855.6	-	529.7, 531.7	-
Ni14CSZ	852.7, -, 855.3	-	529.8, 531.4	-
NiCeZr	852.2, 853.5, 855.4	885.0, 898.3	529.6, 531.3	9
NiCe4CSZ	851.9, 853.2, 855.2	884.7, 898.2	529.4, 531.0	19
NiCe8YSZ	851.9, 853.2, 855.1	885.2, 898.2	529.7, 531.7	34
NiCe14CSZ	852.1, 853.4, 855.5	884.7, 898.3	529.4, 531.1	18

**Table 5:** Particle size results from electron microscopy analysis. For a better comparison, results from H_2_ chemisorption and XRD line broadening are also included.

Sample	Particle Size from H_2_ Chemisorption (nm)	Crystallite Size from XRD (nm)	$\bar{d}$(nm)	${\bar{d}}_{sa}$(nm)	D (%) ^1^
NiZr	27	24	14	27	5.2
Ni4CSZ	16	17	14	20	6.4
Ni8YSZ	32	19	9	23	5.4
Ni14CSZ	38	21	13	20	6.4
NiCe4CSZ	26	16	14	21	5.9

^1^ From HAADF-STEM analysis.

**Table 6:** Results from catalytic experiments.

Sample	Conversion (%)	Selectivity (%)				Products Ratio		H_2_ Yield (%)
		H_2_	CO_2_	CO	CH_4_	H_2_/CO_2_	CO_2_/CO	
NiZr	48	72.9	20.2	4.6	2.2	3.6	4.4	40
Ni4CSZ	75	73.9	23.9	0.2	2.1	3.1	119.5	64
Ni8YSZ	46	75.5	21.5	1.5	1.5	3.5	14.3	36
Ni14CSZ	63	73.8	23.7	0.5	2.1	3.1	47.4	46
NiCeZr	40	76.8	20.5	1.8	0.9	3.7	11.4	34
NiCe4CSZ	68	73.8	23.4	0.6	2.2	3.2	39.0	57
NiCe8YSZ	54	74.1	21.3	2.2	2.5	3.5	9.7	40
NiCe14CSZ	44	75.7	21.6	1.9	0.9	3.5	11.4	33

## Supplementary Materials

The following are available online at https://www.mdpi.com/2079-4991/9/11/1582/s1, Figure S1: Experimental setup for APR of methanol, Figure S2: N_2_ adsorption-desorption isotherms, Figure S3: CO_2_-TPD profiles showing deconvoluted peaks, Figure S4: Ce 3d XPS spectra, Figure S5: O 1s XPS spectra, Figure S6: HAADF-STEM images and EDS-STEM maps for individual components.
