# Supplementary material for: Catalytic Performance of Ni/CeO2/X-ZrO2 (X = Ca, Y) Catalysts in the Aqueous-Phase Reforming of Methanol
Source: Nanomaterials (Basel). 2019 Nov 8;9(11):1582. doi: 10.3390/nano9111582 (PMC6915671; doi:10.3390/nano9111582)
Supplement: Supplementary file 1 [file nanomaterials-09-01582-s001.pdf]

Catalytic Performance of $Ni/CeO_2/X\text{-}ZrO_2$ (X = Ca, Y) Catalysts in the Aqueous-Phase Reforming of Methanol

Daniel Goma [1,2], Juan José Delgado [1,2], Leon Lefferts [3], Jimmy Faria [3], José Juan Calvino [1,2] and Miguel Ángel Cauqui [1,2,*]

1 Departamento de Ciencia de los Materiales e Ingeniería Metalúrgica y Química Inorgánica, Universidad de Cádiz, Puerto Real 11510, Spain; dani.gomajimenez@gm.uca.es (D.G.); juanjose.delgado@uca.es (J.J.D.); jose.calvino@uca.es (J.J.C.)
2 IMEYMAT, Instituto de Microscopía Electrónica y Materiales, Puerto Real 11510, Spain
3 Catalytic Processes and Materials group, University of Twente, P.O. Box 217, 7500 AE Enschede, The Netherlands; l.lefferts@utwente.nl (L.L.); j.a.fariaalbanese@utwente.nl (J.F.)
* Correspondence: miguelangel.cauqui@uca.es; Tel.: +34-956012747

Supporting Information

Table S1. Composition from ICP (%w/w) of the fresh and used catalysts.

Catalysts	Before reaction					After reaction				
	Ni	Ce	Ca	Y	Zr	Ni	Ce	Ca	Y	Zr
NiZr	7.0±0.1	--	--	--	60.0±0.6	7.1±0.1	--	--	--	58.0±0.6
NiCeZr	5.9±0.1	12.6±0.2	--	--	48.4±0.1	5.6±0.1	13.1±0.2	--	--	46.2±0.1
Ni4CSZ	5.0±0.1	--	1.2±0.1	--	60.0±0.6	5.2±0.1	--	1.1±0.1	--	59.8±0.6
NiCe4CSZ	5.9±0.2	13.0±0.1	1.8±0.1	--	47.6±0.3	6.1±0.2	14.0±0.1	1.6±0.1	--	48.4±0.3
Ni8YSZ	5.6±0.2	--	--	4.3±0.1	56.4±0.3	5.8±0.2	--	--	4.1±0.1	57.2±0.3
NiCe8YSZ	5.5±0.1	12.7±0.1	--	3.6±0.1	46.1±0.2	5.5±0.1	13.0±0.1	--	3.9±0.1	48.2±0.2
Ni14CSZ	6.9±0.1	--	4.9±0.1	--	53.0±0.3	6.7±0.1	--	4.6±0.1	--	57.1±0.3
NiCe14CSZ	5.5±0.2	13.5±0.2	4.0±0.1	--	43.3±0.6	5.6±0.2	13.2±0.2	3.8±0.1	--	43.5±0.6

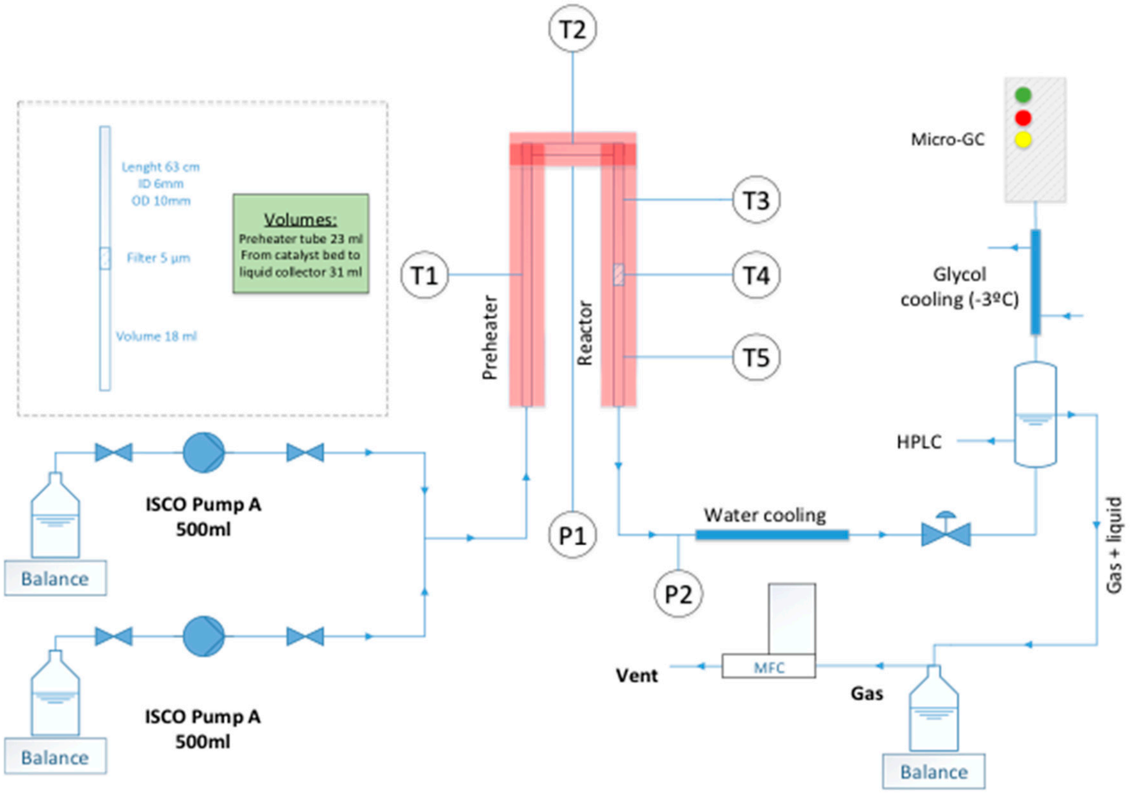

Figure S1. Experimental setup for APR of methanol.

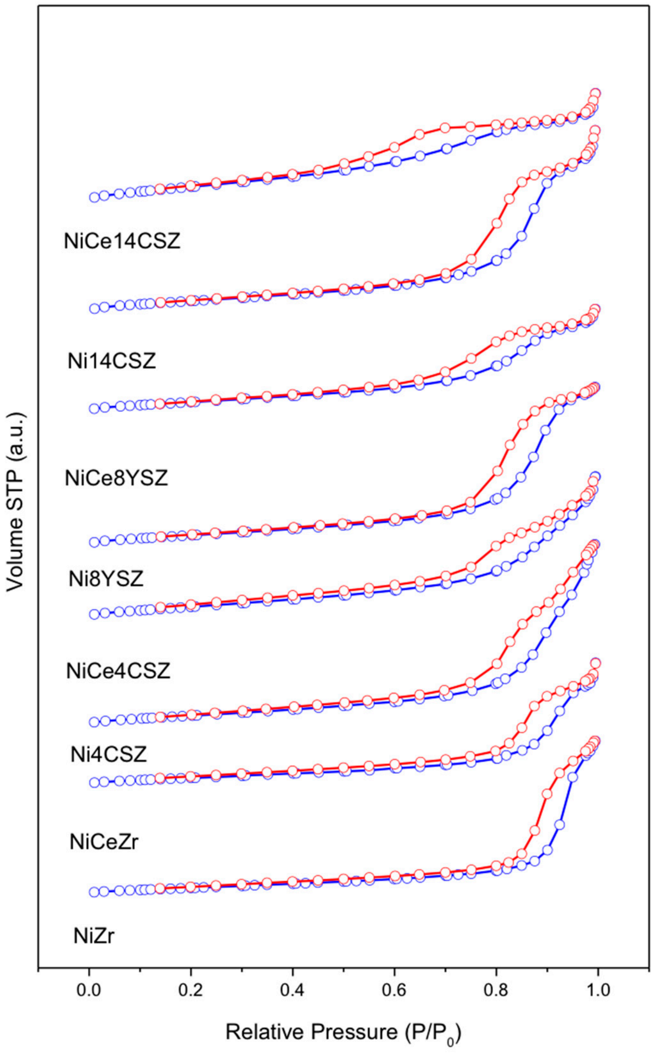

Figure S2. N_2 isotherms.

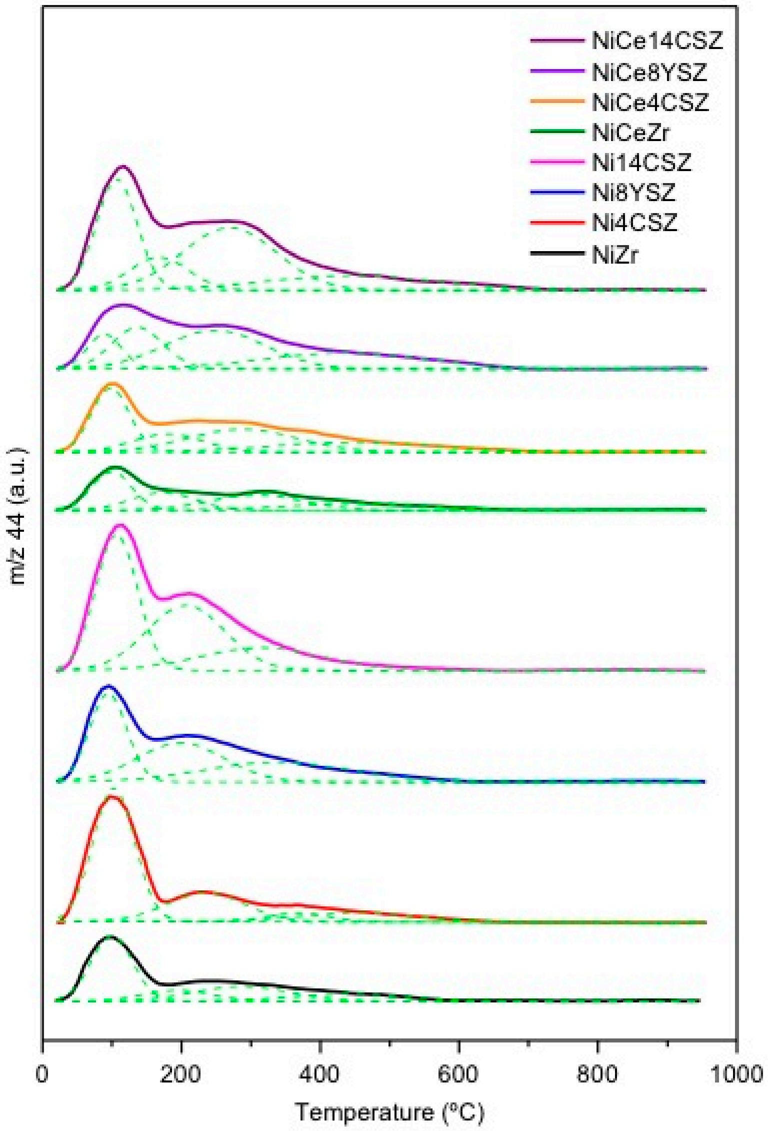

Figure S3. CO_2-TPD profiles showing deconvoluted peaks.

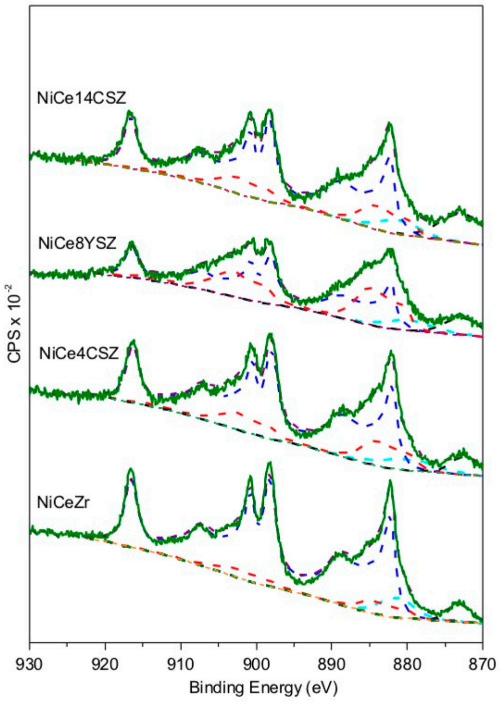

Figure S4. Ce 3d XPS spectra.

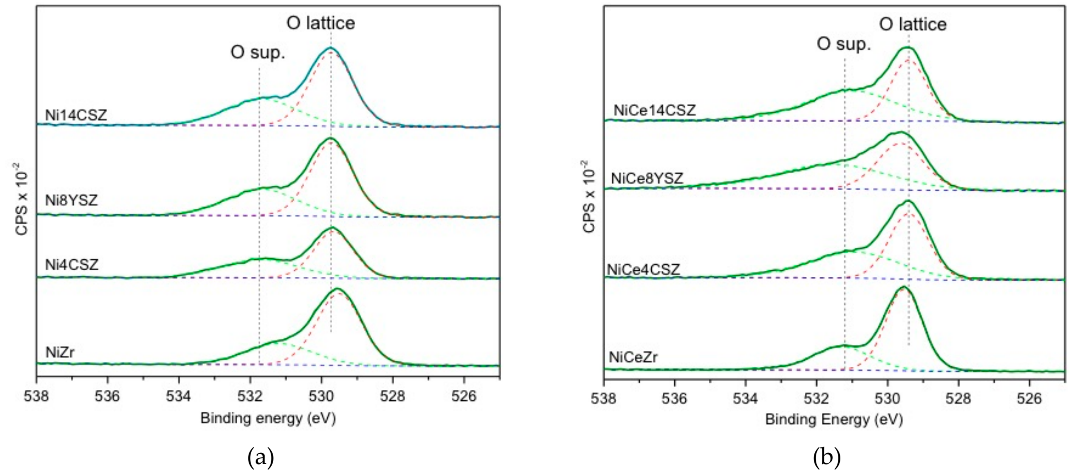

Figure S5. O 1s XPS spectra: **(a)** Samples without Ce; **(b)** Samples with Ce.

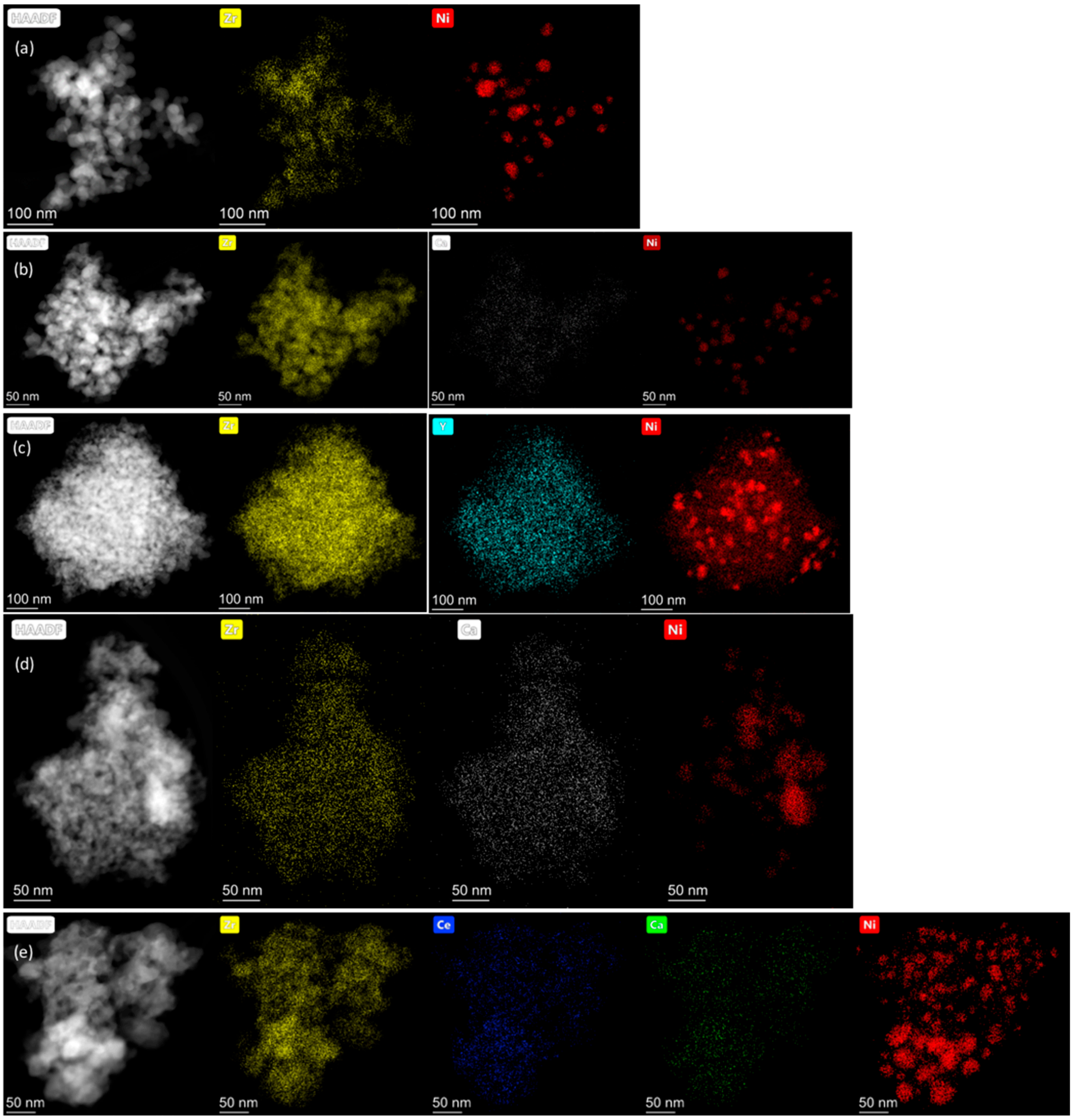

Figure S6. HAADF images and EDS maps for individual components: (a) NiZr, (b) Ni4CSZ, (c) Ni8YSZ, (d) Ni14CSZ and (e) NiCe4CSZ
